# Methods for Streamlining Intervention Fidelity Checklists: An Example from the Chronic Disease Self-Management Program

**DOI:** 10.3389/fpubh.2014.00294

**Published:** 2015-04-27

**Authors:** SangNam Ahn, Matthew Lee Smith, Mary Altpeter, Basia Belza, Lindsey Post, Marcia G. Ory

**Affiliations:** ^1^Division of Health Systems Management and Policy, School of Public Health, The University of Memphis, Memphis, TN, USA; ^2^Department of Health Promotion and Community Health Sciences, Texas A&M Health Science Center, School of Public Health, College Station, TX, USA; ^3^Department of Health Promotion and Behavior, College of Public Health, The University of Georgia, Athens, GA, USA; ^4^Center for Health Promotion and Disease Prevention, The University of North Carolina at Chapel Hill, Chapel Hill, NC, USA; ^5^School of Nursing, Biobehavioral Nursing and Health Systems, The University of Washington, Seattle, WA, USA

**Keywords:** intervention fidelity, quality assurance, Chronic Disease Self-Management Program, aging, evidence-based programs, expert consensus

## Abstract

Maintaining intervention fidelity should be part of any programmatic quality assurance (QA) plan and is often a licensure requirement. However, fidelity checklists designed by original program developers are often lengthy, which makes compliance difficult once programs become widely disseminated in the field. As a case example, we used Stanford’s original Chronic Disease Self-Management Program (CDSMP) fidelity checklist of 157 items to demonstrate heuristic procedures for generating shorter fidelity checklists. Using an expert consensus approach, we sought feedback from active master trainers registered with the Stanford University Patient Education Research Center about which items were most essential to, and also feasible for, assessing fidelity. We conducted three sequential surveys and one expert group-teleconference call. Three versions of the fidelity checklist were created using different statistical and methodological criteria. In a final group-teleconference call with seven national experts, there was unanimous agreement that all three final versions (e.g., a 34-item version, a 20-item version, and a 12-item version) should be made available because the purpose and resources for administering a checklist might vary from one setting to another. This study highlights the methodology used to generate shorter versions of a fidelity checklist, which has potential to inform future QA efforts for this and other evidence-based programs (EBP) for older adults delivered in community settings. With CDSMP and other EBP, it is important to differentiate between program fidelity as mandated by program developers for licensure, and intervention fidelity tools for providing an “at-a-glance” snapshot of the level of compliance to selected program indicators.

## Introduction

Chronic conditions have received nationwide attention because of their adverse impact on individuals’ daily functioning, social interaction, and self-reported quality of life (1) as well as their association with rising healthcare costs (2). Self-management has been viewed as a key factor enabling patients to deal with the everyday challenges of chronic conditions through medical, emotional, and/or role management (3, 4). Despite national calls for more attention to public health strategies that empower Americans to be more involved in their own health (5), many Americans do not inherently possess the skills for actively engaging in self-management behaviors that can help ameliorate the effects of living with chronic diseases.

Evidence-based programs such as the Chronic Disease Self-Management Program (CDSMP) have gained national and international recognition for helping people with chronic conditions learn self-management skills (6, 7). While positive outcomes of CDSMP have been well documented (8), less is known about the actual implementation processes at the state or national level other than gross indicators of program completion or adherence to recommendations regarding class size (9, 10). Thus, the primary purpose of this project was to address intervention fidelity and describe a methodological approach to streamline a fidelity checklist. As a heuristic example, we used the fidelity checklist contained within the CDSMP Fidelity Toolkit (11). A secondary purpose of this project was to use this methodological process to evoke expert opinions about how leaders in the aging services field view the fidelity and quality assurance (QA) processes. As a note, we will consistently use “intervention” fidelity throughout the current study because we focus on a fidelity checklist assessing processes beyond the program itself (e.g., training, before the program, after the program, and evaluation).

### Public health initiatives and evaluations

The U.S. Administration on Aging (AoA) Evidence-Based Disease Prevention Grant Programs, initiated in 2003, have stimulated the development and implementation of evidence-based programs (EBP) for seniors, which dispel earlier myths that health promotion efforts were futile in older populations (12). With this greater national appreciation for the potential of evidence-based health promotion programing for improving health and functioning among older adults, the research questions have shifted from “do we know what works?” to “can we do what is known to work?” This change in focus is now seen with service providers having ready access to a growing list of EBP, which have been widely tested in community and clinical settings and within the aging services network (13, 14).

Yet, the translation of scientifically tested research findings to community-based programs is often slow, fragmented, and subject to speculation by the practitioner community (15). As more EBP are offered by diverse host agencies in more diverse communities, evidence is mounting that their successful dissemination occurs sporadically (15–18). Translational research is coming of age, and models such as RE-AIM are being formulated to serve as guiding frameworks for planning implementation efforts and evaluating the public health impacts of EBP (19–21). More specifically, the RE-AIM framework seeks to identify and overcome the challenges facing program planners and practitioners when moving an EBP from the research setting in which it was developed to the less-than-perfect, resource-limited, and real-world practice environment (19–22).

The RE-AIM framework contains the following five key elements: reach, effectiveness, adoption, implementation, and maintenance (21, 23). Some studies examine all five elements, while others examine outcomes using one or two key elements (16). Our current study focuses on the “I” in RE-AIM, program implementation processes, specifically fidelity monitoring, which can be neglected because of funding and logistic issues in large-scale community-based disease prevention efforts for older adults.

### Intervention fidelity

As EBP become widely disseminated, there has been growing attention to program fidelity in implementation science (16). In terms of translational research, there has been a strong programmatic emphasis on fidelity, which can be defined as the adherence of actual treatment delivery to the protocol originally developed (24). A breach in intervention fidelity, defined as the adherent and competent delivery of an intervention by the interventionists (e.g., trainers, course leaders, and program coordinators) as set forth in the intervention manual (25), threatens licensure and makes it difficult to interpret study results. For example, if the program is not delivered as intended, it is difficult to know if the resulting health outcomes can be attributed to receipt of the intervention or to some other variation in the intervention’s delivery (26).

While maintaining fidelity during program implementation is essential, ensuring the feasibility of monitoring fidelity is also important, especially for organizations with relatively limited capacity to administer the intervention (27). Fidelity to treatment or intervention delivery is one subset of overall treatment fidelity (28) and has often been monitored through observation, interviews, self-assessed fidelity checklists, and pairing of trained facilitators (26). Recent articles have highlighted the importance of having high resource commitment to better monitor fidelity in evidence-based health promotion programs such as CDSMP (29) or EnhanceFitness (30).

### Strategies for ensuring CDSMP program fidelity

Programmatic adherence to implementation aspects of CDSMP is supported by a centralized training and certification system that provides for scripted small-group and participatory workshops (2.5 h a week for 6 consecutive weeks) focused on self-management strategies that provide medical, emotional, and role management skills (4). In regard to the training and certification system, it is noted that there are three hierarchical levels of trainers (31). First, a person can be a certified (lay) leader when she or he completes 4-day Leader Training and facilitates one 6-week workshop within 12 months from the training date. Second, master trainer certification can be obtained when a person completes 4.5-day Master Training and facilitates two 6-week workshops within 12 months of completion of training. Third, a person can be a T-Trainer when she or he completes 4.5-day apprenticeship under supervision of a Stanford approved certifying T-Trainer and conducts at least one Master Training within 12 months completion. In addition, the program coordinator is another important workforce member who plays a key role in implementing CDSMP. The program coordinator, who may be a master trainer or lay leader, typically engages in a variety of tasks such as: identifying community partners, recruiting and supervising workshop leaders and participants, arranging for workshop sites, monitoring intervention fidelity, and evaluating program processes and outcomes (32).

A standardized resource material (e.g., the program guide “Living a Healthy Life with Chronic Conditions” revised in 2012) (4) helps provide general guidance behind the theory and activities. An implementation manual provides more detailed guidance to trainers (33), and a fidelity manual outlines mandatory program requirements. The 2010 CDSMP Fidelity Toolkit (11) contains a fidelity checklist with key aspects listed in the following link: http://patienteducation.stanford.edu/licensing/Fidelity_ToolKit2010.pdf. This fidelity checklist as part of the Toolkit is called “Must Do’s Fidelity Checklist” and provides guidance for personnel with regard to the implementation of CDSMP (e.g., program coordinators, leaders, master trainers, or T-trainers). Personnel are advised to go through the list and check “Yes” for all the items they are currently doing, and are encouraged to incorporate these items with their fidelity plan for the future if they are not able to implement the entire fidelity task right away. These 157 items are chronologically categorized under 7 headings (16 subheadings or blocks): (1) personnel; (2) delivery before training; (3) fidelity during training; (4) fidelity after training; (5) fidelity during workshops; (6) fidelity for leaders and master trainer retention; and (7) fidelity after workshops. Each heading was further divided into a couple of subheadings.

Implementing EBP can require detailed monitoring and tracking information, placing substantial administrative burdens on program coordinators (15). As such, shorter fidelity tools were developed by some states implementing CDSMP including Missouri^1^ and New Jersey^2^. However, none of these fidelity tools was systematically tested. In our role as technical advisors to the AoA Evidence-Based Disease Prevention Grant Programs, we were asked to explore methods for streamlining a fidelity checklist (e.g., CDSMP) and use these methodological processes to seek expert opinions about how leaders in the aging services field view the fidelity and QA processes.

## Materials and Methods

The expert consensus method refers to a multi-phase approach for statistically analyzing pooled opinions that minimizes biases inherent in other systems of summarizing expert viewpoints (34, 35). We used this method to gather and analyze expert opinion on streamlining the 157-item CDSMP fidelity checklist in 2010–2011. We employed three rounds of data collection using Qualtrics software (36) to streamline the original 157-item CDSMP fidelity checklist without losing essential fidelity items but improving feasibility of administration. As a final effort, we held a telephone conference to solicit expert opinion for making final recommendations regarding the use of fidelity checklists. Table 1 displays these four rounds of checklist streamlining and the number of items remaining after each round. Human Subjects approval was obtained from the Texas A&M University Institutional Review Board.

**Table 1:** **Four rounds to streamline CDSMP fidelity checklists (original 157 items)**.

Round	Methods	Reduction criteria	Number of participants	Number of items left
One	Survey	Items related to master trainer training (T-training)/language literacy	114 master trainers	148
		Items that did not meet either statistical or practical approaches	114 master trainers	116
Two	Survey	Items not ranked “very high” in “feasibility” step and not selected as part of a predetermined number of items in “endorsement” step	47 master trainers	34/20/12
Three	Survey	Items not related to their perceptions about the most critical items for assessing program fidelity	7 experts	34
Four	Conference call	Version selection based on organizational resources	7 experts	34^a^/20/12

*^a^Most preferred*.

### Participants

As the credentialing unit, the Stanford University Patient Education Research Center compiles a list of all CDSMP master trainers and manages a listserv for exchange of information. Using this distribution list, we invited all active master trainers in 2010 to participate in our study. In Round One, we collected 119 responses (114 master trainers, 5 others), where 5 responses were eliminated from the final analysis since they were not master trainers. Twenty-six out of 114 were both master trainers and program coordinators. Out of these 114 master trainers, 47 agreed to participate in the second survey. In Round Two, 24 out of 47 master trainers responded to the survey (51% response rate). From this group, nine master trainers were willing to respond to the third survey. In Round Three, seven out of nine master trainers responded to the third survey (78% response rate). In Round Four, eight out of nine master trainers from Round Three agreed to participate in a telephone conference to obtain feedback, advice, and concerns from their experiences delivering and overseeing programs. In this last round, seven of out of eight experts were able to join the group-teleconference call with the study team. Each of the four rounds to streamline the CDSMP fidelity checklist contained multiple steps as shown below.

### Round one: Streamlining the original CSDMP by “ensuring” the overall fidelity of CDSMP

#### Survey process

The survey in Round One was conducted in November and December 2010 with master trainers (*n* = 114) who identified through the Stanford CDSMP master trainer listserv. These experts helped us identify the items in the original 157-item CDSMP fidelity checklist, which they believed to be most important to ensuring the overall fidelity of the program. Participants were asked to rank statements on a scale of one (least important) to five (most important). During this initial step, we eliminated 8 items related to master trainer training and another item referring to language literacy, which resulted in an initial portfolio of 148 unique items to consider. To obtain the most relevant information from participants, the survey included skip patterns that presented participants with a list of items most appropriate to them based on their roles (i.e., master trainers or master trainers/program coordinators). Because of their universal relevance, some items were presented to every group.

#### Reduction criteria

To streamline the CDSMP fidelity checklist, we used both statistical and practical approaches based upon participants’ ranking of each checklist item. First, the statistical approach involved eliminating items based on their distance from the mean score. Because of testing the statistical significance of multiple comparisons, we used the Bonferroni technique by adjusting the significance level (0.05) to avoid the risk of Type I error (37). Second, the practical version involved selecting only those responses that were rated as four or five in terms of importance. We eliminated any items that “failed” to meet criteria for either the statistical or practical cut-offs. For instance, there were 26 items in a question block (or subheading) asking the importance of fidelity before lay leader training. Relying on the statistical approach, the mean of these items was 4.15, and the adjusted significance level for this question block was 0.0019 (i.e., 0.05÷26). This process helped us identify six items that showed significantly lower importance ratings relative to the mean after adjusting multiple comparisons. In the same question block, the practical approach helped us identify another six items that were not rated as four or five. Of the 12 items identified for elimination, 2 items were mutually exclusive and 5 items were common to both the statistical and practical approaches. Thus, we were able to eliminate seven items that failed to meet the statistical or practical cutoff. In other question blocks (e.g., fidelity during workshops), we continued to use both approaches to eliminate items that failed to meet the statistical criteria or the practical criteria and we were able to delete 32 (22%) out of 148 checklist items. At the conclusion of Round One, we asked for volunteers to complete a second survey used to continue this expert consensus process.

### Round two: Streamlining the original CDSMP by ascertaining “feasibility” of administration and “endorsement” of predetermined number of items

#### Survey process

The second survey was conducted in June 2011. In this round, we solicited the opinions of the 47 master trainers who agreed to participate after Round One. Round Two involved asking participants to provide feedback via a two-step process that assessed both “feasibility” and “endorsement.” The first step asked master trainers to rank how feasible they think it is to monitor each fidelity checklist item. Feasibility response categories included “not at all,” “somewhat,” and “very high.” Based on the items identified as having “very high” feasibility in the first step, the master trainers were then asked to select a predetermined number of items (total 34) from each question block that they would endorse for inclusion in the final, shorter fidelity checklist versions. We had 24 master trainers respond to our second survey, resulting in a 51% response rate.

#### Reduction criteria

Employing the practical methodology based on the feasibility and endorsement responses, we were able to generate a shorter Fidelity-12 checklist (12 items), a medium-length Fidelity-20 checklist (20 items), and a longer version Fidelity-34 checklist (34 items). First, we calculated the percent of participants who endorsed each item relative to the total number of endorsements received for each question block (using a scale from 0 to 100%). We also calculated the percent of participants who endorsed each item received relative to the total number of feasibility ratings of “very high” (using a scale from 0 to 100%). The aggregate of this process was assessed on a combined scale of 0–200%. Fidelity-34 included 34 items that received the highest aggregate numbers of each question block (1, 2, or 3 items based on number of items in each question block). Due to our desire to streamline the checklist further, we selected only items with scores of 90 and 100 as cut-points on the combined 0–200 scale. Fidelity-20 (90 as a cut-off) included 20 items while Fidelity-12 (100 as a cut-off) included 12 items. At the conclusion of Round Two, 9 out of 24 master trainers agreed to participate in the survey in Round Three.

### Round three: Finalizing the shortened CDSMP fidelity checklists

We surveyed nine experts to review the three shortened versions of the fidelity checklist and to help us address some remaining questions related to their perceptions about the most critical items for assessing intervention fidelity using Fidelity-34 as the referent. We had seven experts respond to the short survey, and the response rate was 78%. A report of findings was prepared indicating that Fidelity-34 was the one that experts felt best balanced the inclusion of key fidelity items with the feasibility of administration.

### Round four: Confirming the necessity of a shorter CDSMP fidelity checklist

We held a telephone conference with seven experts identified in Round Three to report on our findings and solicit expert opinion regarding the use of fidelity checklists. This conference call was critically important for obtaining feedback and advice from those with experience in delivering and overseeing programs. The experts confirmed a preference for Fidelity-34, but felt that the other two shorter versions should be available since the purpose and resources for administering the fidelity checklist might vary from one setting to another.

### Expert consensus results: Summary recommendations

Table 2 displays the three versions of the CDSMP fidelity checklist (i.e., Fideity-12, Fidelity-20, and Fidelity-34). To sum up these streamlining processes, we included 14 question blocks out of the original 16 blocks (subheadings) that related to personnel to administer and the chronological tasks of CDSMP. In total, 36 items were included in three versions of fidelity checklist. Among these 36 items, 2 items were only included in the Fidelity-20 (“Have all Leaders facilitate at least once a year,” and “Have trainees participate in two practice teaching activities during training”). Just like Fidelity-34, Fidelity-20 included at least one item of each question block. However, Fidelity-12, as the shortest version of CDSMP fidelity checklist, did not include any items from four question blocks: “Fidelity after lay leader training;” “Fidelity before master training;” “Fidelity when counseling out leaders/master trainers during training;” and “Fidelity during workshops: Physical environment and material resources.” Although Fidelity-34 was highly recommended in Rounds Three and Four, CDSMP experts from the telephone conference also recommended providing organizational partners with all three versions and allowing them to select the best checklist based on their resources. For instance, organizations with time restraints and limited staffing may prefer shorter checklist versions to the longer version, whereas organizations with better staffing and time resources may want to utilize the more thorough version of the checklist.

**Table 2:** **Streamlined fidelity checklists using expert consensus method: a CDSMP case study^a^**.

	Checklist version		
	Fidelity-12	Fidelity-20	Fidelity-34
**Question block #1: program coordinator qualifications**			
Ql They are very familiar with both the program fidelity and program implementation manuals	X	X	X
Q2 They have observed a Leader or Master Training	X	X	X
**Question block #2: lay leader qualifications**			
Q3 They are not afraid to speak in front of groups	**–**	**–**	X
Q4 They read, write, and speak the language of the workshop participants	X	X	X
Q5 They are able to attend all 4 days of training and complete two practice teachings during training as prospective leaders	X	X	X
**Question block #3: fidelity before lay leader training**			
Q6 Apply for, renew, or confirm receipt of organization’s program license^b^	**–**	**–**	X
Q7 Adhere to recommended schedule for leader trainings (total of 4 days: recommended 2 days per week for 2 weeks)	**–**	X	X
Q8 Have two certified master trainers who are committed to conduct entire training sessions	X	X	X
Q9 Inform participants that their full attendance and participation is required on all training	**–**	**–**	X
Q10 Prepare a complete leader’s manual for each participant	**–**	**–**	X
**Question block #4: fidelity after lay leader training**			
Q11 Have all leaders facilitate at least once a year	**–**	X	**–**
Q12 Not let those leaders with whom there are concerns facilitate workshops^b^	**–**	X	X
**Question block #5: master trainer qualifications**			
Q13 They are willing and available to attend a 4.5-day-master training	X	X	X
Q14 They either have led two workshops as a leader either before coming to master training or are willing and available to lead two workshops within 1 year after master training	**–**	**–**	X
**Question block #6: fidelity before master training**			
Q15 Prepare master trainer manuals and leader manuals for each participant	**–**	**–**	X
Q16 Determine the most recent training materials are being used for training (most current version are 3rd edition, living a healthier life with chronic conditions book and CDSMP manual (2006)	**–**	**–**	X
Q17 Follow the Stanford Patient Education Research Center’s checklist for master trainings (obtained upon confirmation of training request)	**–**	**–**	X
Q18 Inform participants their full attendance and participation is required on all training days	**–**	X	X
**Question block #7: fidelity during master training**			
Q19 Have trainees participate in two practice teaching activities during training	**–**	X	**–**
Q20 Have trainees complete the second practice teaching session and demonstrate a minimum set of core competency as observed by a master trainer or T-trainer^b^	X	X	X
Q21 Make sure that training must be at least 27 h, usually over 4.5 days	**–**	**–**	X
Q22 Have training offered by two certified T-trainers	**–**	**–**	X
Q23 Understand and agree with the importance of program fidelity	**–**	**–**	X
**Question block #8: fidelity after master training**			
Q24 Conduct one leader training a year^b^	X	X	X
**Question block #9: fidelity in judging trainee competence during training**			
Q25 Adheres to the curriculum (also includes appropriate presentation of charts)	X	X	X
Q26 Facilitates group contributions particularly in the following types of activities: brainstorming, action planning, action plan feedback, and problem solving	**–**	X	X
Q27 Models activities appropriately	**–**	**–**	X
**Question block #10: fidelity when counseling out leaders/master trainers during training**			
Q28 Observe and document problem behaviors	**–**	**–**	X
Q29 Give the trainee specific reasons and examples of why they are concerned	**–**	**–**	X
Q30 Tell the trainee what she/he did well, but also tell her/him clearly how they are expected to improve	**–**	**–**	X
**Question block #11: fidelity for lay leader and master trainer retention**			
Q31 Have defined protocols for resolution of potential personality conflicts, communication problems, improper behavior with participants and co-leaders or co-trainers is in place	X	X	X
**Question block #12: fidelity during workshops: physical environment and material resources**			
Q32 Have the necessary number and quality of educational materials and supplies	**–**	**–**	X
Q33 Offer the workshop 2.5 h a week over 6 weeks	**–**	X	X
**Question block #13: fidelity during workshops: lay leader performance**			
Q34 Have two leaders teach the workshops	X	X	X
Q35 Ensure that leaders use facilitation techniques appropriately and effectively	**–**	X	X
**Question block #14: fidelity after workshops**			
Q36 Track leader activity (i.e., programs they teach, retention rates)^b^	X	X	X

*X, include; –, exclude*.

*^a^CDSMP sites must comply with licensure and fidelity requirements as defined by Stanford University Patient Education Research Center. The purpose of this research was to demonstrate the use of expert consensus method to streamline intervention fidelity monitoring checklists and to improve monitoring of evidence-based program fidelity. For the most current CDSMP licensure information, please visit Stanford University Patient Education Research Center (http://patienteducation.stanford.edu/licensing/)*.

*^b^Some of these items represent a slight modification from the Stanford Self-Management Fidelity ToolKit (2010) to fit the current question format*.

## Discussion

Using the original CDSMP fidelity checklist as a case example, this research provides a methodology for streamlining fidelity checklists that have many unique items, making field implementation resource-intensive and challenging. We see the methodology described in this paper as our key contribution, which can be applied to different EBPs. It should be noted that these shortened checklists are for research only and require further field-testing before specific endorsements can be made. The CDSMP fidelity checklist has been updated since the time of this study, and a new fidelity manual was developed that stresses the importance of setting intervention fidelity within an overall fidelity plan. These updated materials also distinguish “must do” fidelity strategies from those that are “nice to do” to strengthen program fidelity (33). For the most current requirements, please refer to the Stanford University Patient Education Center website^3^.

Applying the expert consensus technique, we consolidated the 157-item CDSMP fidelity checklist into three abbreviated versions without sacrificing fidelity items deemed essential by master trainers. Due to its overall length, the original Stanford checklist was often used more as a self-assessment reminder rather than an actual fidelity checklist. Given the importance of program implementation as a core component of program evaluation (21, 23), we believe shorter fidelity checklists will prove beneficial to current and future program leaders and coordinators who are trying to implement EBP with limited financial or time resources.

Though the abbreviated checklists are likely less time-consuming than the original version, further improvements may be needed. First, any checklist should be seen as just one element in an overall QA plan (38). Additionally, one might explore different delivery modalities. For example, enabling users to access fidelity checklists online could further enhance their usefulness. An online monitoring system would allow for real-time review and feedback so that program coordinators and trainers can evaluate their progress and fidelity as implementation is occurring, making changes when necessary to adhere to program guidelines.

In addition to generating three abbreviated versions of CDSMP fidelity checklist, this study also demonstrated an effective use of expert consensus method for generating consensus from a broad field of CDSMP experts with varying experience and perspectives. When dealing with a diversity of opinions, problems may arise due to conflicting viewpoints, self-censorship due to lack of anonymity, incomplete feedback loops or poor communication, or lack of defined statistical methods for attributing quantitative values to subjective factors. Many of these potential pitfalls were avoided by using three iterative rounds of online surveys. Because respondents did not communicate directly, they were free to express their opinions. Statistical and practical methods were used to give each respondent’s perspective equal weight in reaching consensus. In the Round Four telephone conference, expert participants did directly communicate, but because much of the work related to consolidating the list was already concluded, the participants were able to reach consensus easily.

At the end of the process, we were able to identify the most relevant and applicable items and garner experts’ endorsement of the abbreviated checklists as useful. The methods employed in this study could be used as a model for administrators of other EBP aiming to reduce the length of a fidelity checklist for program monitoring. However, it is important to note that CDSMP sites must comply with licensure requirements and be familiar with the official CDSMP Implementation and Fidelity Manuals, which will need to be updated on a regular basis, if programmatic or licensure requirements change.

Several limitations should be considered. First, our relying on small-group processes should be understood for its lack of true representation of the general CDSMP master trainer population. Nevertheless, we believe the current study contributes to the literature related to streamlining a fidelity checklist because of the relative large sample size (*n* = 114) in Round One survey, and multiple rounds of survey, and a final telephone conference with content experts. Second, our study is potentially limited by focusing on one major stakeholder group, i.e., master trainers. The inclusion of various opinions from CDSMP completers, lay leaders, T-trainers, program developers, and researchers in academia in the Round Three survey could have ensured better representation by various CDSMP implementers and stakeholders. Third, the CDSMP program was revamped after the initiation of this study. While some aspects of the program have been improved upon, we believe the basic elements have remained consistent to ensuring fundamental intervention fidelity based on what is needed before, during, and after workshop delivery. Hence, the need for shortened fidelity checklists is still relevant. Fourth, the study was conducted within a specific time period when federal funding was available for CDSMP implementation. Such changes in the availability of resources and program demands may influence, which fidelity checklist items are most feasible to monitor. However, the ever-changing context and limited funding resources might make three short versions of the CDSMP fidelity checklist helpful and valuable. Fifth, there were noticeable dropouts between streamlining rounds. Since data were not collected from the purposive list of participants, it was not possible to make real comparisons between responders and non-responders. Sixth, we note that generating one intervention fidelity tool may not be the most effective or efficient way to capture all aspects of fidelity, which could be implementation fidelity for leaders or program fidelity for program coordinators and agency administrators. Last, we do not know the level of expertise and knowledge among participated master trainers since we did not collect this information. Nevertheless, we strongly believe master trainers are appropriate experts given the required level of training and experience with delivering CDSMP. To retain their certificates, master trainers are required to conduct 4-day Leader training within 18 months of original training and conduct either a 4-day Leader Training, a 6-week series of community workshops, or a Leader cross training (31).

## Conclusion

Fidelity is critical to the successful dissemination of EBP. The challenge for the field is balancing resources for program delivery with those for assuring intervention fidelity. In this study we explore one avenue for reducing the administrative resources needed for maintaining fidelity. The study demonstrates the importance of finding ways to streamline intervention fidelity checklists for EBP, suggesting several key points. First, an expert consensus method is a viable approach to assessing the usability of fidelity checklists. Second, online software (e.g., Qualtrics) can be used for efficient data collection, analyses, and tracking of participant response with built-in reminder systems. Third, it is important to have input from stakeholders with various roles to have a comprehensive picture of intervention fidelity. We encourage researchers to apply this expert consensus model to other EBP, and to conduct further study of the reliability, validity, and practicability of fidelity checklists.

## Conflict of Interest Statement

The authors declare that the research was conducted in the absence of any commercial or financial relationships that could be construed as a potential conflict of interest.

This paper is included in the Research Topic, “Evidence-Based Programming for Older Adults.” This Research Topic received partial funding from multiple government and private organizations/agencies; however, the views, findings, and conclusions in these articles are those of the authors and do not necessarily represent the official position of these organizations/agencies. All papers published in the Research Topic received peer review from members of the Frontiers in Public Health (Public Health Education and Promotion section) panel of Review Editors. Because this Research Topic represents work closely associated with a nationwide evidence-based movement in the US, many of the authors and/or Review Editors may have worked together previously in some fashion. Review Editors were purposively selected based on their expertise with evaluation and/or evidence-based programming for older adults. Review Editors were independent of named authors on any given article published in this volume.

## Footnotes

^1^http://www.ncoa.org/chamodules/documents/MOCDSMPQITool.pdf

^2^http://www.ncoa.org/chamodules/documents/NJ_PeerLeaderChecklist.doc

^3^http://patienteducation.stanford.edu/licensing/
